# Hybrid effectiveness-implementation study of two novel spectrally engineered lighting interventions for shiftworkers on a high-security watchfloor

**DOI:** 10.1093/sleepadvances/zpad051

**Published:** 2023-11-21

**Authors:** Sara C Bessman, Elizabeth M Harrison, Alexandra P Easterling, Michelle N Snider, Sebastian M M Preilipper, Gena L Glickman

**Affiliations:** Henry M. Jackson Foundation for the Advancement of Military Medicine, Inc. (HJF), Bethesda, USA; Department of Psychiatry, Uniformed Services University of the Health Sciences, Bethesda, USA; Henry M. Jackson Foundation for the Advancement of Military Medicine, Inc. (HJF), Bethesda, USA; Department of Psychiatry, Uniformed Services University of the Health Sciences, Bethesda, USA; Henry M. Jackson Foundation for the Advancement of Military Medicine, Inc. (HJF), Bethesda, USA; Department of Psychiatry, Uniformed Services University of the Health Sciences, Bethesda, USA; Henry M. Jackson Foundation for the Advancement of Military Medicine, Inc. (HJF), Bethesda, USA; Department of Psychiatry, Uniformed Services University of the Health Sciences, Bethesda, USA; Henry M. Jackson Foundation for the Advancement of Military Medicine, Inc. (HJF), Bethesda, USA; Department of Psychiatry, Uniformed Services University of the Health Sciences, Bethesda, USA; Department of Psychiatry, Uniformed Services University of the Health Sciences, Bethesda, USA

**Keywords:** circadian, light, melanopic, spectrum, alertness, sleep, shiftwork, intervention, implementation

## Abstract

Shiftwork leads to myriad negative health and safety outcomes. Lighting countermeasures can benefit shiftworkers via physiological effects of light (e.g. alerting, circadian adjustment), and short-wavelength light is the most potent for eliciting those responses; however, limited work indicates it may not be required for alerting. We developed similar-appearing light boxes (correlated color temperature: 3000–3375 K; photopic illuminance: 260–296 lux), enriched (SW+, melanopic EDI: 294 lux) or attenuated (SW-, melanopic EDI: 103 lux) in short-wavelength energy, and implemented them on a high-security watchfloor. Efficacy and feasibility of these two novel lighting interventions were assessed in personnel working 12-hour night shifts (*n* = 47) in this within-participants, crossover study. For each intervention condition, light boxes were arranged across the front of the watchfloor and illuminated the entire shift; blue-blocking glasses were worn post-shift and before sleep; and sleep masks were used while sleeping. Comparisons between baseline and intervention conditions included alertness, sleep, mood, quality of life (QOL), and implementation measures. On-shift alertness (Karolinska Sleepiness Scale) increased in SW- compared to baseline, while changes in SW+ were more limited. Under SW+, both mood and sleep improved. Psychomotor vigilance task performance did not vary by condition; however, perceived performance and QOL were higher, and reported caffeine consumption and sleep onset latency were lower, under SW-. For both interventions, satisfaction and comfort were high, and fewer symptoms and negative feelings were reported. The addition of spectrally engineered lights to this unique work environment improved sleep, alertness, and mood without compromising visual comfort and satisfaction. This paper is part of the Sleep and Circadian Rhythms: Management of Fatigue in Occupational Settings Collection.

**Statement of Significance**

With the high prevalence and necessity of shiftwork in our society, interventions that mitigate its harmful effects on health and safety are warranted. A leading countermeasure is light, which acts via a range of physiological effects, including circadian resetting, alerting properties, and mood enhancement. Most lighting interventions employ off-the-shelf lighting products and rarely assess implementation. In this study, two light boxes were spectrally engineered to appear similar but have different physiological effects. Additionally, intervention feasibility and implementation were assessed. Both interventions were well-received and differentially improved outcomes for night shiftworkers relative to their typical lighting. Future studies should include less restrictive work environments and different shiftworker populations as well as employ enhanced measures of individual photic exposure patterns and circadian phase.

## Introduction

While shiftwork is necessary and prevalent in our 24-hour society, nonstandard schedules have been associated with a host of negative health and safety consequences [1, 2]. Ideally, work times are aligned both with the circadian timing system, which generates endogenous rhythms with a period of approximately 24 hours (e.g. sleep–wake patterns), and with the solar clock, by which the circadian system is entrained to a more precise 24-hour day. Yet, shiftworkers often work and sleep during times that are not aligned with either, increasing the risk of accidents, errors, and injuries [1], and leading to sleep disruption, which can itself impair health and performance [3]. Furthermore, waking and sleeping at the wrong time can independently lead to circadian misalignment, as light serves to shift the circadian clock. In combination, these factors have compounding negative impacts on performance and safety, and are associated with other unwanted health outcomes, such as metabolic disorders, cardiovascular disease, immune function impairment, and psychological health issues [1]. Considering the ubiquity and necessity of shiftwork, there is a need for interventions that can help mitigate its many harmful effects.

One of the leading countermeasures shown to improve health and performance in shiftworkers is light [4–9]. Historically, architectural lighting in most workplaces has been designed to support visual task performance; however, light also has myriad physiological effects that are distinct from vision, including phase-shifting of circadian rhythms, increasing alertness, and enhancing mood [10]. The mechanism by which light elicits these responses has been fairly well-described and is primarily mediated via melanopsin-containing intrinsically photosensitive retinal ganglion cells (ipRGCs) [11–16]. Photic input is conveyed through the eye along the retinohypothalamic tract to the suprachiasmatic nucleus (SCN) in the hypothalamus, which serves as the central circadian pacemaker and regulates downstream rhythms. In addition, ipRGCs project directly to other non-visual nuclei and regulatory centers of the brain, bypassing the SCN, which suggests that certain physiological effects of light may be regulated without pacemaker involvement [17–20]. Finally, light information also follows a separate visual pathway via rod and cone photoreceptors [10]. Importantly, the same light will provide input along all of these pathways, to both visual and non-visual systems; thus, lighting interventions for shiftworkers should be designed to enhance circadian health and alertness while also supporting visual performance.

In developing lighting countermeasures for shiftworkers, it is important to consider the physical properties of light that can influence physiological responses, including timing, intensity, and spectrum [9, 10, 21]. Many workplace lighting interventions employ pulses from light boxes, with timing often based on convenience (e.g. light in break rooms) [6, 22–27]. Yet, in order to best support circadian entrainment, the timing of photic exposure is key, with light early in the biological night delaying the circadian clock to a later time, and light later in the biological night advancing it earlier [28–30]. Thus, the beginning and end of the biological night (e.g. “dawn” and “dusk”) are likely to be of particular importance [31]. Theoretically, this may be especially true for those on inverted schedules, where day/night contrast in light input might be the most important element for artificially constructing a new “day” and “night.” While phase-shifting is the light response most impacted by the timing of photic exposure, other non-visual physiological effects of light also vary by circadian phase. For example, limited work on the alerting effects of light, though largely studied at night when baseline alertness is naturally low and increases can be detected, has shown time-of-day effects in healthy participants on standard schedules [32, 33].

Even at the same biological time, different photic intensities and wavelengths will elicit differential responses to light. Specifically, across various physiological effects of light, studies have demonstrated a characteristic dose–response, with increasing intensities of light resulting in greater responses. Importantly, there is a threshold photic intensity required to obtain any effect, and a point of saturation at which higher intensities will not yield a greater magnitude response [32, 34–37]. Human studies under highly controlled conditions demonstrate a dose–response to white fluorescent light, with a half-saturation of ~90–125 lux and a range of responsiveness across ~2.5 orders of magnitude for melatonin suppression, phase-shifting, and alerting effects of light [36, 38]. The dose–response function will shift depending on spectrum, and at a given intensity (that is not below threshold or above saturation), short-wavelength light is the most potent across various non-visual physiological functions and may have a longer lasting impact as well [33, 39], with the greatest sensitivity to ~480 nm light as compared to all other tested wavelengths [32, 34, 40–45]. Yet, architectural lighting typically tends toward moderate intensities, warmer color temperatures, and less short-wavelength emissions, which is not best for circadian health and safety in workplace settings [46]. A metric quantifying the biological potency of light for non-visual responses, termed melanopic equivalent daylight illuminance, or melanopic EDI (in lux), takes both intensity and spectral sensitivity into account [47]. Recent consensus-based recommendations suggest exposures of melanopic EDI < 1 lux, < 10 lux and > 250 lux to be optimal for night, evening, and day, respectively [48].

Studies employing monochromatic light have been critical for enhancing our understanding of the physiological mechanisms underlying photic responses, though polychromatic white light is best for most practical applications, as maintaining visual performance and color discrimination remains important. Consequently, white-appearing lights with varied proportions of short-wavelength energy have been developed and tested under highly controlled conditions; however, that work has yielded somewhat mixed results. In one laboratory study, for example, high color temperature (17 000 K) short-wavelength-enriched (133 melanopic lux) polychromatic light during the biological night suppressed plasma melatonin and improved subjective alertness relative to lower temperature white light (4000 K, 79 melanopic lux). Yet, there were no effects on circadian phase or performance [49]. In a separate study, filtering short-wavelength light resulted in melatonin suppression similar to the dim control comparison, while performance was enhanced just as much as the unfiltered white light condition [50]. Considering that similar doses of light can both suppress melatonin and phase-shift rhythms [36], those findings raised the possibility of developing a lighting intervention for shiftworkers that can improve cognitive performance with little effect on the timing of circadian rhythms and sleep, which could be particularly beneficial to those working rotating shift schedules. Taken together, these basic science studies help to inform the development of lighting interventions for shiftworkers.

Biologically potent daytime light exposure with relatively darker nights is a basic principle that has been translated for shiftworker applications and yielded promising results. Studies that simulate night shiftwork are generally robust, with carefully timed light exposures facilitating circadian readjustment in participants, as assessed via melatonin, body temperature, and sleep [51]. For studies in actual shiftworkers, in addition to the less controlled conditions, the broad distribution of circadian phases and chronotypes, slow rate of adjustment (~1 hour/day), and off-shift light exposure (reverting to diurnal phase on days off, exposure to bright sunlight upon leaving work), all contribute to variability in data and make it impossible to identify a single lighting paradigm that works equally well for all [10, 21, 52]. In spite of this, bright white light administered in the workplace has been shown to improve alertness, performance, mood, quality of life (QOL), and sleep [4, 8, 53, 54]. Sleep and alertness may be further improved when photic input is reduced after the end of the shift upon leaving work in the morning and/or during sleep [4, 7, 55–57]. Based on these benefits and the potential for dissociable effects [50], lighting countermeasures for shiftworkers may be tailored to target specific, and even differential, responses. Furthermore, new evidence-based metrics [47], with practical guidance for both daytime and nighttime levels [16], provide a model by which specific doses that are theoretically more reflective of biological potency can be selected, measured, and tested. Finally, advances in technology now allow for the development of lighting interventions that simultaneously prioritize and support vision, health, and subjective experience, which is critical for implementation and uptake.

**Figure 1.**
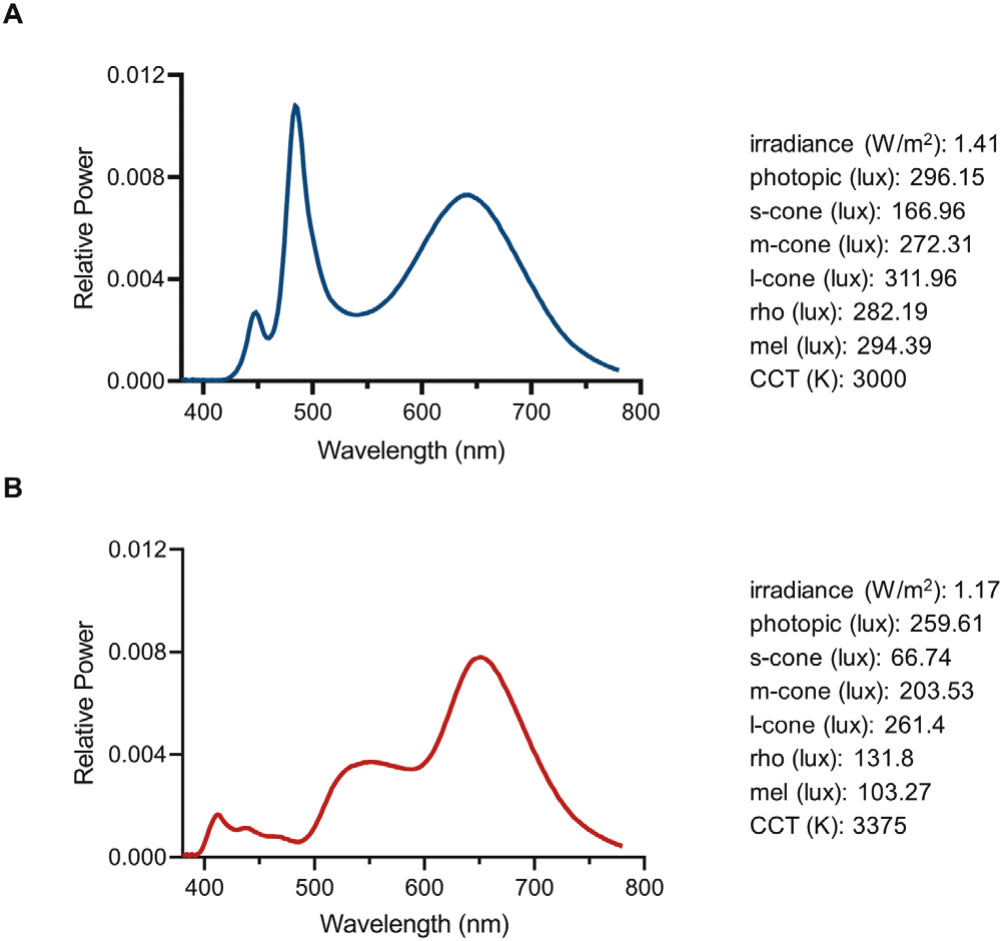
Spectrally engineered lightboxes. Spectral power distributions are provided for (A) SW+ and (B) SW- light boxes, along with associated irradiance, alpha-opic equivalent daytime illuminances (e.g. rhodopsin [rho] and melanopic [mel]), and correlated color temperature for each.

For this study, we developed two types of light boxes that were spectrally engineered to contain near-cone metameric polychromatic white stimuli, either enriched in the short-wavelength region of the visual spectrum (SW+), or attenuated in that same region (SW-) (see spectral power distributions, SPDs, in Figure 1). Metameric polychromatic stimuli are perceived as the same color and luminance but with distinct SPDs and thus, different biological potencies (as measured via melanopic EDI). The near-cone metameric lights used in this study were designed to minimize disparities in both visual task performance and participant expectations while resulting in significantly different melanopic EDIs (above and below the recommended daytime levels), aiming to elicit differential physiological responses (see Figure 1). In combination with the light boxes, which provided additional illumination in the immediate work space, blue-blocking glasses and sleep masks were used to reduce photic input after work hours and on days off, prior to and during sleep, for both SW+ and SW- lighting intervention conditions.

The two novel lighting interventions were implemented, and alertness, sleep, mood, and QOL were assessed, in active duty service members working 12-hour night shifts on a high-security watchfloor. We hypothesized that SW+ would elicit broad physiological effects (e.g. changes in alertness, sleep, and mood) whereas SW- would enhance alertness while minimizing alterations in circadian rhythms (e.g. delays in sleep). In addition to our primary outcomes, implementation measures (e.g. participant satisfaction, barriers and facilitators to uptake, and planned future use) were included in the design of this study for each component of the two intervention conditions: the light boxes, the blue-blocking glasses, and the sleep masks. As implementation science is a relatively young field, applied studies of shiftworker interventions have seldom included such measures [58, 59]. Yet, implementation outcomes are vital for understanding why (or why not) certain elements of an intervention are taken up by participants, which directly impacts practical efficacy [60]. Because of these considerations, we utilized a hybrid effectiveness-implementation design [61] to assess both the efficacy and feasibility of these two distinct light-based shiftworker countermeasures.

## Methods

### Participants

Participants were 59 active duty service members working 12-hour night shifts on a shoreside submarine watchfloor at Naval Support Activity Hampton Roads in Norfolk, VA. Data were collected between July 4 and September 9, 2021. Most individuals completed their study participation within a 48-day period (two participants took slightly longer—52 and 64 days). Participants were required to remain on 12-hour shifts for the duration of the study in order to be included. Two participants withdrew from the study due to schedule changes, and six participants worked 6- or 8-hour shifts (rather than 12 hours) in one or more conditions and thus, were not included. If participants took medications for sleep or alertness, criteria specified that they must have been on the medication for ≥6 weeks and planned to continue the medication throughout the study. Exclusion criteria included medical conditions for which light treatment would be contraindicated, such as a history of bipolar disorder and/or significant ocular health problems; however, no volunteers needed to be excluded for any of these reasons.

### Light boxes

There were 16 light boxes engineered (BIOS Lighting, Carlsbad, CA, USA), with half enhanced in the short-wavelength region of the spectrum to create a biologically potent signal (SW+; Figure 1A), and half attenuated in the short-wavelength region in order to promote alertness without additional circadian effects (SW-; Figure 1B). Light boxes measured 66.04 cm × 66.04 cm × 17.78 cm, with a 3 m grounded power cord (110V), and were composed of an array of white-appearing light emitting diodes. Vertical illuminance and spectral measurements were taken in complete darkness using a SpectraScan Spectroradiometer PR-670 (PhotoResearch, North Syracuse, NY, USA) that was held stationary, centered, and positioned 50.80 cm from the surface of the light boxes with a tripod, and alpha-opic equivalent daytime illuminances were later derived using the Commission Internationale de l’Éclairage toolbox (CIE S 026/E:2018). SW+ and SW- each contained a different spectral composition and included the following specifications, respectively: CCT (3000 K/3375 K), photopic illuminance (296 lux/260 lux), melanopic EDI (294 lux/103 lux); for both types, ≤-0.1% UV (see Figure 1 for further details).

### Schedules, protocol, and workplace setting

**Figure 2.**
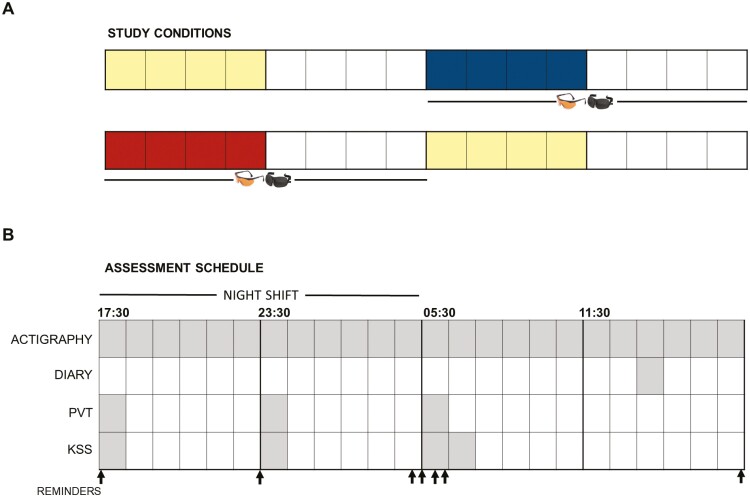
Study protocol. (A) An overview of the two, 16-day intervention periods for a sample participant who had their first baseline assessment prior to SW+ and their second baseline assessment after SW- is represented in this diagram. Each box represents 1 day, including baseline night shifts (yellow), days off (white), SW+ night shifts (blue), and SW- night shifts (red). Blue-blocking glasses and sleep masks were worn during intervention weeks only. The 16-32-day washout period is not shown. (B) The schedule of assessment for the study is illustrated for the second work night in a series of night shifts. Throughout the baseline and intervention conditions, an actigraph watch was worn continuously, and a sleep diary was completed daily. Psychomotor vigilance task (PVT) and Karolinska Sleepiness Scale (KSS) assessments occurred near the beginning, middle, and end of the night shift, with an additional KSS completed immediately after the shift. Email and/or text message reminders are depicted with arrows and were sent throughout.

The watchfloor was organized into four, ~15-member teams on staggered, rotating schedules where only one team was on shift at any given time. Night shifts started at 17:30 with a few exceptions (two individuals worked 18:00–06:00). Schedules followed a 16-day cycle, where individuals worked a series of 4 shifts of the same shift type (e.g. 4 night shifts) followed by 4 days off (creating an 8-day work week), followed by a second set of 4 night shifts and 4 days off (a second 8-day work week). At the end of the 16 days, they switched to the opposite shift type and repeated the cycle. So, if they performed night shifts on their previous cycle, then the upcoming cycle would consist of only day shifts, with four on, four off, four on, four off. The majority of participants completed two, 16-day data collection periods that were designed to coincide with their existing 16-day night work schedule cycles—the first period containing an 8-day baseline and 8-day SW+ light intervention, and the second containing an 8-day baseline and 8-day SW- light intervention (Figure 2A; interventions described below). The order of baseline and intervention conditions within those 16-day periods was pseudo-randomized, resulting in a 16–32-day washout period in between interventions, depending on assigned order; half the participants received conditions in one order (Baseline, SW+, SW-, Baseline), while the other half received them in another (SW+, Baseline, Baseline, SW-).

All recruitment and data collection activities were conducted by researchers at the Uniformed Services University and approved by the Institutional Review Board (USUHS.2019-026). In the weeks leading up to the study, watchfloor staff were provided with a study flyer and informed that study staff would be onsite to meet with everyone and answer questions. Consenting took place across 2 days in order to capture all four teams during morning and evening shift turnover times. Immediately after signing the consent form, participants were each given a tote bag that contained written instructions, a study calendar, a sleep diary, an actigraph watch (Actiwatch Spectrum Plus, Philips, Murrysville, PA, USA) with charger, a sleep mask (Dream Essentials Contoured Sleep Mask; East Hartford, CT, USA), and blue-blocking glasses (UVEX SCT-Orange; Honeywell, Morris Plains, NJ, USA; Ultra-Spec 2000 S0360X model for those with corrective lenses (*n* = 15), Skyper S1933X model for those without). Participants were instructed on all study activities, and told to wear the actigraph watch continuously during data collection and charge it when asked (Figure 2B). They were also instructed to use the blue-blocking glasses and sleep masks during intervention weeks only. An SMS-based system was used to send participants text and/or email message reminders throughout data collection; phones were not allowed on the watchfloor, so on-shift reminders occurred via work email.

**Figure 3.**
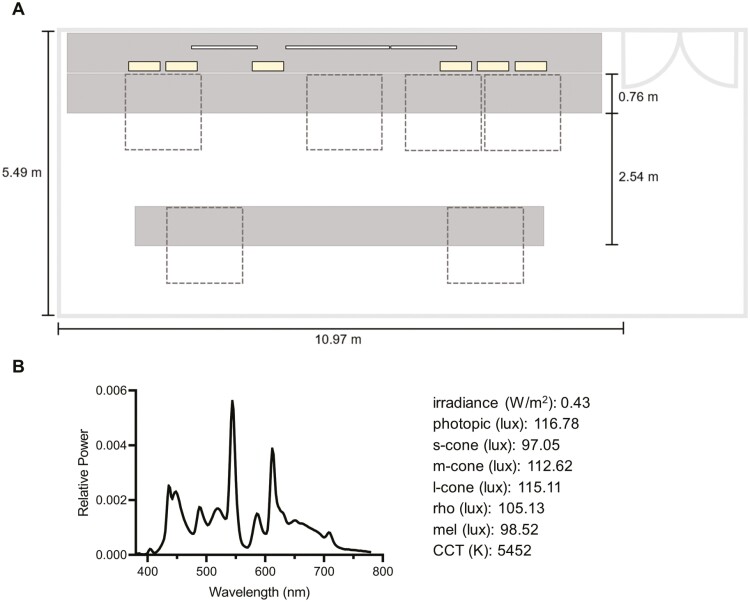
Watchfloor schematic and baseline light. (A) This diagram represents a schematic of the watchfloor space, including key dimensions. The front of the space contained a row of three oversized monitors (thin white rectangles) suspended well above the surface upon which six of either SW+ or SW- light boxes were arranged underneath, unobstructed, in the same configuration (thicker yellow rectangles), all facing into the room. The front row of four workstations (dashed boxes) is where the vast majority of participants spent most of their time; two additional workstations were located behind those, and all workstations directly faced the oversized monitors (which required regular attention of participants) and lightboxes. In total, there were ~45 computer and television monitors under the control of watchfloor staff that varied somewhat in size, intensity, and color temperature; only the three oversized ones are depicted here. (B) The spectral power distribution as well as irradiance, alpha-opic equivalent daytime illuminances (e.g. rhodopsin [rho] and melanopic [mel]), and correlated color temperature are averages of measurements taken for baseline conditions from the front ledge of the desk at the front row of workstations from a seated position at eye-level, with the meter directed toward the center of the nearest lightbox (all turned off) and avoiding a direct line with any surrounding monitors.

The watchfloor is a sensitive compartmented information facility (SCIF) that prohibits the use of all recording, photographic, and other electronic media devices. Access required approvals, proper levels of clearance and/or escorts, and sanitization (i.e. watchfloor staff turning off some displays and monitors, and removing sensitive documents prior to study staff entering the space). There were no windows, and the space was illuminated by 17 overhead fluorescent lights as well as ~45 computer and television monitors of various sizes. During the study period, six light boxes were set up along the front counter of the watchfloor and directed into the main space (see Figure 3A). The SW+ light boxes were in place from July 4—August 5, 2021, followed by SW- from August 5—September 9, 2021, and light boxes were present but not turned on under baseline conditions. During the intervention weeks, each light box was connected to a timer that was scheduled to automatically turn the lights on and off at night shift start and end times, respectively; this was regularly confirmed by study staff either in person or via communication with onsite leadership. Additionally, there was a smaller SCIF located across the hall from the main watchfloor where six participants were stationed (~1–2 from each team), with two individuals on shift at a time, following the same schedule as the main watchfloor. As with the main watchfloor space, there were no windows, and this space was illuminated by seven overhead fluorescent lights as well as computer and TV monitors of various sizes. Two light boxes were set up side-by-side on a table, 246.38 cm in front of the two primary workstations, at the same time as the main watchfloor light boxes.

Light measurements in the main watchfloor and smaller SCIF were both taken with the SpectraScan Spectroradiometer PR-670 (PhotoResearch, North Syracuse, NY), and alpha-opic equivalent daytime illuminances were later derived using the Commission Internationale de l’Éclairage toolbox (CIE S 026/E:2018). For the main watchfloor, measurements were taken at each workstation depicted in Figure 3A, and for the smaller space, measurements were taken from the two primary workstations (as described above). When taking measurements, a direct line with workstation monitors was avoided, in order to capture light from all sources: overheads, monitors, and light boxes. In addition, the sensor meter was positioned at the front ledge of each workstation desk and directed toward the center of the closest light box (for both baseline and intervention conditions). Vertical illuminance and spectral power distributions were obtained at approximate eye-level from both seated and standing positions for a standardized height (106.68 cm and 163.50 cm from the floor). Since the vast majority of participants spent most work time seated in the front workstations of the main watchfloor space, the average of these measures is reported. Average baseline lighting specifications for the main watchfloor are described in detail in Figure 3B. The smaller SCIF had comparable specifications at baseline, with a photopic illuminance of 230.31 lux and a melanopic EDI of 132.50 lux. Average light levels for both intervention conditions (including usual overhead lighting and light boxes) were a photopic illuminance of 327.89 lux and a melanopic EDI of 321.31 lux under SW+, and a photopic illuminance of 286.37 lux and a melanopic EDI of 156.45 lux for SW-. The estimated percent time spent in the work space was noted in daily diaries; however, the precise location within the space varied between (and to a much lesser degree, within) individuals.

### Outcome measures

#### Alertness.

At the beginning, middle, and end of each night shift, participants completed a Karolinska Sleepiness Scale (KSS) [62] to assess alertness on a scale from 1 to 9, which was completed by hand and time-stamped (on paper, since electronic media devices were not authorized in the SCIF). Immediately upon filling out each paper KSS slip, the paper was deposited into a box on the watchfloor. Forty-five minutes after the end of their night shift, participants responded to a modified KSS via text message (“On a scale from 1 to 9, how sleepy are you? (1 = extremely alert, 5 = neither, 9 = fighting sleep)”).

A validated, 3-minute tablet-based psychomotor vigilance task (PVT) (Pulsar Informatics, Philadelphia, PA, USA) was also employed, as it is the current gold standard for the objective measurement of alertness, easy to administer, and highly sensitive to sleep deprivation, time of day, and circadian disruption [63]. Participants performed the PVT at the beginning, middle, and end of the second night shift during each series of 4 night shifts. If participants were unable to complete it that night, they were asked to complete it on the third night instead. For the PVT, three tablets were set to a standardized angle and affixed at standing height to a desk in the main lobby area (a.k.a, “the quarterdeck”), where tablets were permitted for use (outside of the main watchfloor, just inside the building entrance). Because the space did not allow for chairs to be placed at the table, participants were asked to keep a consistent standing position when completing the assessment, as changes in posture can influence results [64]. Participants were instructed on how to complete the PVT during the consent process, and instructions were posted next to each tablet to serve as a reminder. Site visits were performed every ~2 weeks to collect paper KSS data and upload PVT data to a server.

#### Sleep diary and actigraphy.

**Figure 4.**
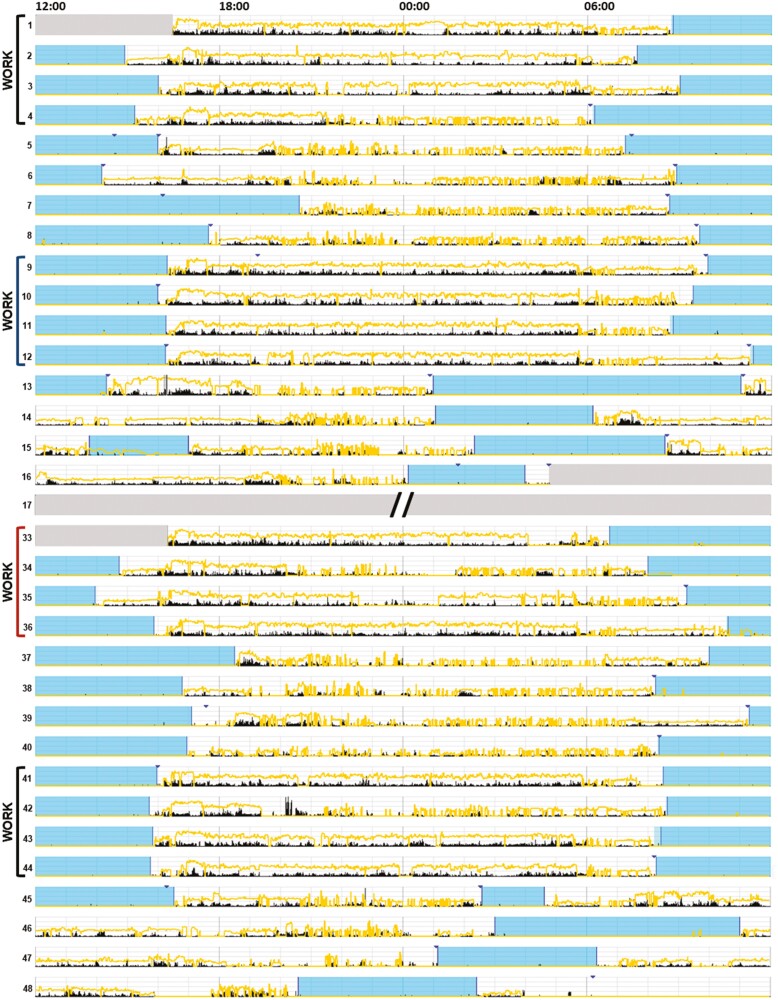
Sample actogram of sleep, activity, and light patterns from a single participant. Days are on the vertical axis, and time of day is on the horizontal axis, with representations of relative timing for activity (black markings), sleep (blue shading), and photopic lux (yellow lines). This particular participant received the study conditions in the following order: Baseline, SW+, SW-, Baseline. The 16-day interval of working day shifts between the two intervention weeks is cropped out of the figure for ease of viewing.

Participants slept at home, and completed a modified version of the Consensus Sleep Diary on a daily basis to track sleep and other behaviors, such as caffeine use throughout the day [65, 66]. Variables of interest were: sleep quality (5-point Likert scale); sleep per 24 hours (daily duration in minutes); sleep onset latency (minutes to fall asleep); wake after sleep onset (WASO, minutes awake after initiating sleep); clock times for sleep onset, mid-sleep and wake; and whether caffeine was used daily, as well as estimated daily amount (in mg). Wrist-worn actigraphs were used to continuously measure activity and photic exposure patterns throughout the study (see Figure 4 for example). Variables of interest included those above, with the exception of sleep quality, and additionally included measures of sleep consolidation: sleep efficiency; average sleep bout (the average length in minutes of each uninterrupted sleep bout); and average wake bout (the average length in minutes of each uninterrupted waking bout). Each variable was analyzed separately for days on versus days off.

#### Questionnaires.

Before baseline, participants completed a ~60-minute online survey (Qualtrics; Provo, UT, USA) containing the reduced Horne-Ostberg Morningness Eveningness Scale [67] to assess chronotype, and questions regarding demographics, work, and circadian and sleep health that came primarily from the survey of shiftworkers (SOS) [68, 69]. At the end of each 8-day work week, participants were prompted to complete a brief, 10-minute online survey (Qualtrics; Provo, UT, USA) consisting of the QOL Index [70], the World Health Organization Health and Performance Questionnaire (HPQ) [71], and original questions about the intervention (see implementation section below). For QOL, participants rate both their satisfaction with, and the importance of, 33 items. One item regarding unemployment was removed from our version (as all participants were employed). Per scoring instructions, the 32 satisfaction responses were normalized, weighted by the 32 importance scores, and then summed to generate one total score, as well as sub-scores in four domains: health and functioning, social and economic, psychological/spiritual, and family. The HPQ was scored according to the absolute scoring method [72], whereby a single item rating of recent self-rated performance (scale 0–10) was considered. The question phrasing was modified slightly to ask about the last week in order to align with intervention duration (the original item inquires about the last 4 months).

#### Intervention dose and implementation.

Individual photic exposure information and experience

Photosensors embedded within the actigraphs were used to measure individual light patterns. Participants also tracked time spent on the watchfloor on each shift (0, 25%, 50%, 75%, or 100%) in daily diaries. On each weekly questionnaire, participants were asked about the following aspects of their lighting experience: perceived satisfaction with, and the comfort and brightness of, the lights, each on a scale of 1–5; symptoms they attribute to the light (check all that apply: glare, headache, nausea, and fatigue); and mood and feelings they attribute to the light (check all that apply: happy, sad, excited, relaxed, sleepy, anxious, wired, and alert). Finally, on their last weekly questionnaire only, participants were asked to endorse which light boxes, if any, they would keep, and to self-rate their alertness with the light boxes compared to without (much better, somewhat better, same, somewhat worse, and much worse).

#### Blue-blocking glasses and sleep masks.

When diaries were completed daily upon awakening from their main sleep, participants recorded whether they used blue-blocking glasses and sleep masks that day, as well as how long (in minutes) and when blue-blocking glasses were worn (never; on your way home (but not while driving); outside before sleep; inside before sleep). Weekly questionnaires during intervention weeks asked participants to report the ease of use and the likelihood of future use of these two at-home intervention components, and they were prompted to explain their answer, as has been done for previous studies [54]. If a participant reported being unlikely to use the intervention components in the future, they were prompted with additional multiple-choice options as to why they were unlikely to use them, including an “Other” option with an additional open-ended response. They were also asked to rate their sleep with blue-blocking glasses and sleep masks relative to without.

### Data analysis

Missing data were expected in this field study, and the number of participants in each analysis varies. For all measures, participants had to have sufficient data in at least one baseline and one intervention condition to be included, and also meet criteria for inclusion in more than one dataset (e.g. both diary and KSS). While most individuals participated in both baseline conditions (*n* = 35), to maximize sample size for each outcome, data from the first baseline condition were used when available, and data from the second baseline condition were used when no data from the first baseline were available.

For the on-shift KSS and PVT, which were completed across the work shift, time-on-shift was binned into three categories (17:00–20:29, 20:30–02:29, and 02:30–06:15 for the beginning, middle, and end of shift, respectively). For the KSS, which was completed on all work shifts, time-on-shift was included as an additional fixed factor. Post-shift sleepiness scores were analyzed separately, as they were delivered via a different modality (text), and it was unknown what activities participants were engaging in at the time of response; only scores received within 2 hours of the time in which the prompt text was sent were included. PVT results from the middle of the shift alone were analyzed, as the tablet was in a high-traffic area due to watchfloor security requirements, and during site visits, distractions appeared to be unacceptably high during these shift transitions. This was confirmed via examination of errors/lapses. In addition, outlier analysis was conducted on PVT lapses for quality control purposes, and three extreme outliers were identified (≥3 interquartile range); associated responses were excluded from analyses of PVT measures. PVT outcomes of interest included lapses, false starts, errors, mean reaction time, speed, efficiency, and aggregate scores.

For all sleep data, transition days (on to off, off to on) were generally excluded, resulting in data from ~3 days of work days and/or ~3 days off per condition per person. Actigraphy scoring was conducted by two team members (APE, EMH) with high inter-rater reliability (97.7%). Datasets were constructed for work days and days off, with no less than 48 hours of data in at least two conditions required for inclusion, and excluding any intervals where clear schedule discrepancies existed (e.g. participant was scheduled to work, but actigraphy clearly indicates otherwise). Whether caffeine was consumed and the number and type of caffeinated drinks were reported daily, and estimates of dose (mg) were derived from either the company’s website or one of the following two sources [73, 74]. For all timing data (sleep and wake times, mid-sleep), averages for work and off days were computed for each individual for each condition and then subjected to vector analyses.

All statistical analyses were carried out in IBM SPSS Statistics for Windows, Version 28.0. (Armonk, NY) with the exception of the vector analyses, which were conducted in R (open source; circular package). Descriptive statistics were calculated for demographics. Age, baseline self-reported workload, and pacing from the SOS, as well as baseline QOL scores and KSS scores across the shift, were examined for participants located in the smaller SCIF compared to those on the main watchfloor via t-test. No differences were found, so all eligible participants from both spaces were analyzed together. Chi-square analyses were used for examination of nominal outcomes (feelings, symptoms). Mixed models with participant as a random factor, condition as a fixed factor (baseline, SW+, and SW-), and sex as a covariate were used for all continuous and ordinal outcome measures of interest, with Bonferroni adjustments for post hoc tests. Chronotype (Supplementary Table S1) was considered as a factor, but no main effects of chronotype on main outcomes of interest were found, thus it was not included in any model; we did, however, replicate previous findings in military samples where, contrary to expectations given the relative young age and predominantly male sample, chronotype tended to skew towards morningness [75, 76].

To address any potential attrition bias, all mixed-model analyses were repeated including only individuals that had completed all three conditions, and while statistical power was reduced, the overall pattern of results remained consistent with results reported below, for all measures. Means, vector lengths (rho), and standard deviations were also calculated for timing data (diary and actigraphy-based sleep, mid-sleep, and wake times), and differences across conditions were examined with Watson–Williams tests. All tests were evaluated at the *p* < 0.05 level, unless otherwise specified.

## Results

In addition to the exclusions mentioned above, four additional participants had insufficient data across conditions (no baseline data and/or no intervention data). The resulting final sample size was 47 individuals (*n* = 8 females; age 28.95 + 0.76 years; see Supplementary Table S1 for participant demographics). A total of 35/47 individuals participated in both of the intervention conditions; nine and three participated in SW+ and SW- only, respectively. On average, participants who completed items relating to percent time spent on the watchfloor/intervention environment (*n* = 41) reported spending ~76% of each work shift on the watchfloor, with no difference across conditions (76%, 76%, and 75% for baseline, SW+, and SW-, respectively; *p* = 0.76). While the precise location of each individual throughout each shift was not tracked, the majority of the workstations were located in the front row (Figure 3A), and during the study team’s site visits, those workstations were used by the vast majority of participants; therefore, most individuals likely received similar exposure patterns overall.

### Alertness

**Figure 5.**
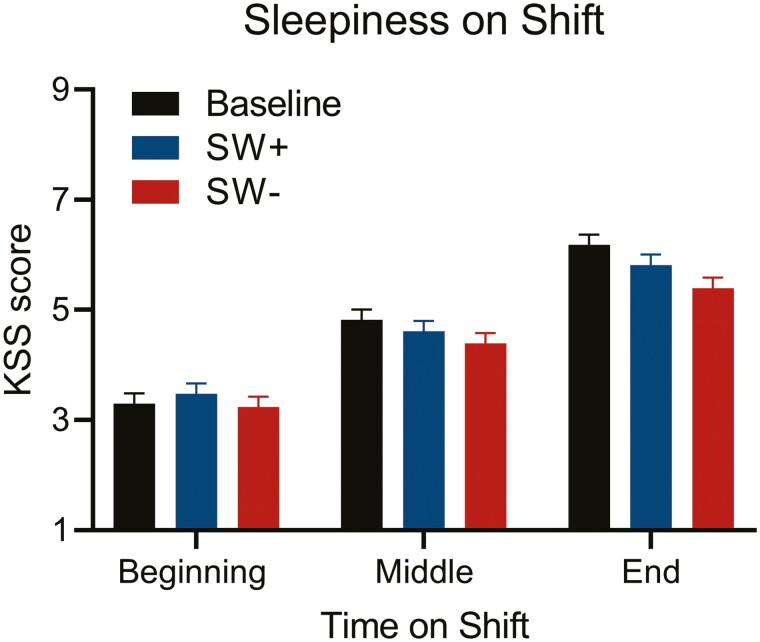
Sleepiness/alertness across the shift and by condition. Estimated marginal mean Karolinska Sleepiness Scale (KSS) scores from participants at the beginning, middle, and end of night shifts (up to 4 nights per person per condition), with bars representing baseline (BL; black), SW+ (blue), and SW- (red) conditions. Sleepiness varied by condition (F_(2,1179.98)_ = 8.60, *p* < 0.001), with lower sleepiness scores in the SW- condition as compared to both BL (*p* < 0.001) and SW+ (*p* < 0.05). Sleepiness also increased across the shift (F_(2,1168.55)_ = 290.87, *p* < 0.001), with lowest values at the beginning of the shift, intermediate in the middle, and highest at the end of the shift (all *p* < 0.001). There was also a significant condition by time-on-shift interaction (F_(4,1168.07)_ = 2.45, *p* < 0.05), likely driven by the changing relationship between SW+ and BL scores across the shift.

**Figure 6.**
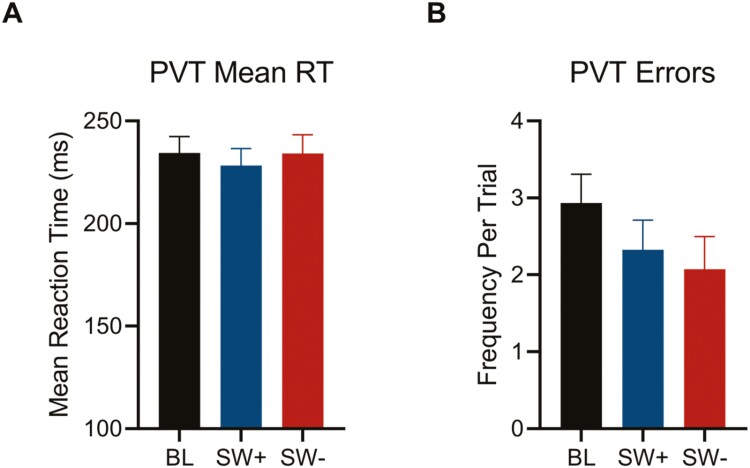
Mid-shift PVT performance across conditions. Estimated marginal means for the psychomotor vigilance task (PVT) (A) mean reaction time and (B) errors are shown for baseline (BL), SW +, and SW-. There were no effects of condition on any PVT variable examined (all *p* > 0.12).

Analyses were conducted on a total of 1220 on-shift sleepiness scores (KSS) (*n* = 45); 298 post-shift sleepiness scores (*n* = 36); and 97 mid-shift PVT responses (*n* = 37). For on-shift sleepiness (Figure 5), there were main effects of both lighting conditions (*p* < 0.001) and time-on-shift (*p* < 0.001), as well as a significant interaction (*p* < 0.05; see Figure 5 legend for full statistics). KSS scores for baseline (mean ± SEM, 4.77 ± 0.16) and SW+ (4.64 ± 0.16) conditions were both higher as compared to the SW- condition (4.34 ± 0.16) (both *p* < 0.05), with no differences between SW+ and baseline conditions (*p* = 0.60). When comparing KSS scores across time on-shift, scores were lowest at the beginning of the shift (3.34 ± 0.16), intermediate in the middle (4.61 ± 0.16), and highest at the end of the shift (5.80 ± 0.16) (all *p* < 0.001). There was no main effect of condition on post-shift KSS scores (6.35 ± 0.17, 6.22 ± 0.18, and 6.34 ± 0.19 for baseline, SW+ and SW- conditions, respectively; F_(2, 271.20)_ = 0.27, *p* = 0.77), and all examined PVT parameters were not statistically different across conditions (all *p* > 0.12; Figure 6). Sex differences were observed in PVT efficiency and speed, with lower values for females (both *p* < 0.05); however, only six female participants contributed to that particular dataset.

### Sleep diary and actigraphy

Work day diary entries were analyzed (n’s range from 41 to 43), and descriptive statistics for each condition are shown in Table 1. Sleep quality varied by condition (*p* < 0.01), with higher sleep quality in the SW+ condition relative to baseline (*p* < 0.01), but SW- did not vary from either other condition (*p* > 0.22). A similar pattern was found for WASO (*p* < 0.05), with less WASO in SW+ compared to baseline (*p* < 0.05) (*p* > 0.18 for all other comparisons). By contrast, sleep onset latency was also different across conditions; however, it was individuals in the SW- condition that had a ~4 minutes shorter sleep onset latency than baseline (*p* < 0.05 for both omnibus and post hoc tests). Similarly, the mean percentage of days on which individuals consumed caffeine was lower in the SW- condition relative to baseline, with an associated lower mean in milligrams as well (*p* < 0.05 for all tests). Diary-based sleep per 24 hours was not different between conditions on work days (*p* = 0.15), nor did mean diary-based sleep, wake, and mid-sleep times vary on work days (*p* ≥ 0.17 for all, see Table 1 and Supplementary Table S2; Figure S1).

**Table 1.** Diary-Based Sleep

Measure	Baseline	SW+	SW-	F	*p*
Work days					
Sleep quality (1–5)	3.10 ± 0.08 ††	3.40 ± 0.09 **	3.28 ± 0.09	5.17	0.006**
Sleep per 24 h	396.89 ± 9.77	388.28 ± 10.67	416.72 ± 10.93	1.92	0.151
Sleep onset latency	16.52 ± 1.63 §	15.02 ± 1.69	12.84 ± 1.72 *	3.29	0.038*
Wake after sleep onset	10.97 ± 2.10 †	4.95 ± 2.23 *	6.86 ± 2.26	4.21	0.016*
Caffeine (Y/N)	58.1% ± 0.06 §	49.0% ± 0.07	45.7% ± 0.07 *	3.90	0.021*
Caffeine (mg)	128.69 ± 18.45 §	114.82 ± 19.08	90.90 ± 19.26 *	3.94	0.021*
Days off					
Sleep quality (1–5)	3.31 ± 0.10	3.58 ± 0.11 §§	3.20 ± 0.12 ††	4.90	0.008**
Sleep per 24 h	473.82 ± 15.73	458.95 ± 16.35	464.26 ± 16.79	0.49	0.616
Sleep onset latency	16.65 ± 2.06	17.67 ± 2.14	17.37 ± 2.23	0.12	0.886
Wake after sleep onset	10.32 ± 4.18	6.64 ± 4.31	14.97 ± 4.42	2.66	0.073
Caffeine (Y/N)	39.8% ± 0.07	39.6% ± 0.07	40.0% ± 0.07	0.00	0.997
Caffeine (mg)	62.24 ± 14.70	69.24 ± 14.10	73.92 ± 14.70	0.72	0.487

Each column lists the mean ± SEM for diary parameters across the three study conditions. Bonferroni-adjusted post hoc tests were used for specific contrasts, with significant differences indicated from baseline (*), SW+ (†), and SW- (§). All units are in minutes, except Sleep Quality (5-point Likert scale; higher is better) and caffeine usage (“mg” represents “milligrams”). Statistics for main effects of study conditions are shown in the final two columns, and asterisks indicate statistical significance. For both post hoc tests and main effects, the number of symbols indicates significance level (1 = *p* < 0.05, 2 = *p* < 0.01, 3 = *p* < 0.001). For “Caffeine (Y/N),” the percentage of days individuals indicated they consumed caffeine (“Yes”) in that condition are shown. Diary-based “sleep per 24 h” is the sum of the duration of the main sleep interval described by the diary, plus the duration of any noted naps. Ns differ by variable and by work days/days off, and range from 32 to 43.

Diary entries were also analyzed for days off (*n*’s range from 32 to 37), and descriptive statistics are shown in Table 1. Even on days off, when participants were not exposed to workplace lighting, sleep quality still varied by condition (*p* < 0.01), with higher sleep quality in the SW+ condition relative to the SW- condition (*p* < 0.01), and a trend for SW+ to be higher than baseline as well (*p* = 0.06). All other comparisons of sleep and caffeine measures on days off were not statistically significant (*p* > 0.07). Mean diary-based bed, wake, and mid-sleep times on days off were also not significantly different between conditions (*p* ≥ 0.64; Supplementary Figure S1 and Table S2).

A total of 39 individuals had sufficient actigraphy data across conditions (Table 2 includes descriptive statistics). An actogram for the whole study from a single participant is shown in Figure 4. For work days, the average sleep bout length was longer in the SW+ condition than both baseline and SW- conditions (*p* < 0.05, for both omnibus and post hoc tests). No other actigraphy-based sleep parameter differed by condition, for either work days or days off (all *p* > 0.12). Mean actigraphy-based bed, wake, and mid-sleep times were statistically similar across conditions for both work days and days off (Supplementary Figure S1 and Table S2). Sex differences were observed in total sleep time (*p* < 0.01), as well as the number of wake and sleep bouts (both *p* < 0.05), with female participants demonstrating relatively shorter, albeit less-fragmented, sleep.

**Table 2.** Actigraphy**-**Based Sleep

Measure	Baseline	SW+	SW-	F	p
Work days					
Sleep efficiency	86.7% ± 0.79	87.7% ± 0.79	86.5% ± 0.86	1.46	0.233
Sleep per 24 h	340.82 ± 11.12	349.46 ± 11.20	347.72 ± 12.24	0.39	0.680
Sleep onset latency	4.97 ± 1.07	6.27 ± 1.08	6.17 ± 1.22	0.64	0.526
Wake after sleep onset	47.90 ± 3.21	44.01 ± 3.23	47.83 ± 3.57	0.93	0.397
Average sleep bout	16.68 ± 0.81	18.56 ± 0.81 §	16.23 ± 0.90 †	4.40	0.013*
Average wake bout	2.07 ± 0.09	2.07 ± 0.09	2.03 ± 0.10	0.17	0.884
Days off					
Sleep efficiency	85.96% ± 0.80	85.50% ± 0.84	85.02% ± 0.96	0.44	0.643
Sleep per 24 h	409.12 ± 17.49	423.44 ± 18.50	389.76 ± 21.23	0.99	0.374
Sleep onset latency	6.61 ± 1.72	6.99 ± 1.85	11.78 ± 2.19	2.09	0.126
Wake after sleep onset	58.04 ± 4.34	62.91 ± 4.54	58.46 ± 5.09	0.68	0.509
Average sleep bout	15.48 ± 0.80	15.74 ± 0.84	15.55 ± 0.94	0.05	0.949
Average wake bout	2.08 ± 0.07	2.13 ± 0.07	2.01 ± 0.08	0.74	0.479

Each column lists the mean ± SEM for actigraphy parameters across the three study conditions. Bonferroni-adjusted post hoc tests were used for specific contrasts, with significant differences indicated from baseline (*), SW+ (†), and SW- (§). All units are in minutes, except sleep efficiency (%). Statistics for main effects of study conditions are shown in the final two columns, and asterisks indicate statistical significance. For both post hoc tests and main effects, the number of symbols indicates significance level (1 = *p* < 0.05, 2 = *p* < 0.01, 3 = *p* < 0.001); *N* = 38 (work days) and 28 (for days off); and “h” represents “hours.”

### QOL and self-rated performance

QOL and self-rated performance results, including means, standard errors, and statistics, are shown in Table 3 (*n* = 40). There was a main effect of condition for the QOL Total score (*p* < 0.05), with post hoc tests indicating higher scores for individuals in the SW- condition relative to baseline (*p* < 0.05), and no differences between other conditions (both *p* > 0.18). Results were similar for the Health subscale (*p* < 0.05 for baseline vs. SW-, *p* > 0.51 for other comparisons). There was also a main effect of condition for the Socioeconomic component (*p* < 0.05), which includes measures relating to one’s job, with post hoc tests indicating higher scores for individuals in the SW- condition relative to SW+ (*p* < 0.05), and no other differences between conditions (both *p* > 0.25). No differences were found for the Psychological/Spiritual or Family subscales (*p* = 0.48 and *p* = 0.08, respectively). Self-rated performance on the HPQ (*n* = 41) was higher for individuals in the SW- condition relative to baseline (*p* < 0.05); all other comparisons were not statistically significant (both *p* > 0.44).

**Table 3.** Quality of Life (QOL) and Self-rated Performance by Condition

Measure	Baseline	SW+	SW-	F	*p*
Total QOL	18.89 ± 0.77 §	19.21 ± 0.78	20.04 ± 0.80 *	3.81	0.027*
Health QOL	18.64 ± 0.83 §	19.35 ± 0.84	20.12 ± 0.87 *	3.53	0.035*
Socioeconomic QOL	18.55 ± 0.70	17.82 ± 0.71 §	19.23 ± 0.73 †	4.54	0.014*
Psychological/Spiritual QOL	18.03 ± 1.03	18.34 ± 1.04	18.71 ± 1.06	0.74	0.482
Family QOL	21.84 ± 0.89	22.74 ± 0.91	23.75 ± 0.97	2.67	0.077
Performance	7.79 ± 0.21 §	8.06 ± 0.28	8.35 ± 0.23 *	5.12	0.022*

Each column lists the mean ± SEM for QOL and self-rated performance across the three study conditions. Bonferroni-adjusted post hoc tests were used for specific contrasts, with significant differences indicated from baseline (*), SW+ (†), and SW- (§). QOL measures are scaled from 0 to 30, and Performance from 0 to 10. Sample sizes are *n* = 41 (baseline), 37 (SW+), and 30 (SW-). Statistics for main effects of study conditions are shown in the final two columns, and asterisks indicate statistical significance. For both post hoc tests and main effects, the number of symbols indicates significance level (1 = *p* < 0.05, 2 = *p* < 0.01, 3 = *p* < 0.001).

### Implementation

#### Light boxes.

**Figure 7.**
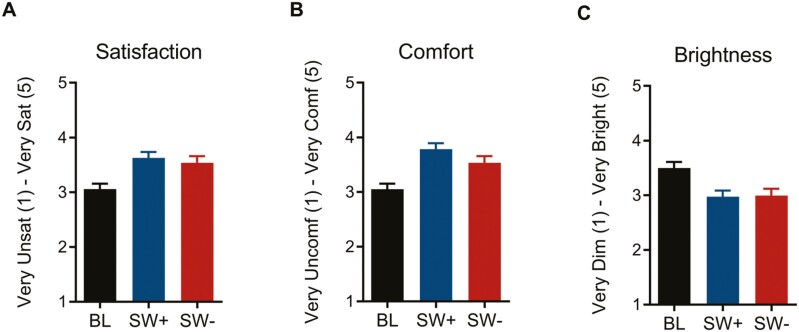
Brightness, comfort, and satisfaction with the light boxes by condition. Estimated marginal means of responses on a five-point scale (1–5) administered in the weekly (every 8 days) questionnaire are shown for baseline (BL), SW+ , and SW-. (A) Participants had higher satisfaction under both intervention conditions than in baseline (F_(2, 69.78)_ = 12.65, *p* < 0.001; *p* < .001 for SW + and *p* < 0.01 for SW-). Additionally, they found (B) the light boxes in both conditions to be less bright (F_(2, 70.07)_ = 7.60, *p* < .001; *p* < 0.01 for both SW+ and SW-), and (C) more comfortable (F_(2, 69.96)_ = 16.06, *p* < .001; *p* < 0.001 for SW + and *p* < 0.01 for SW-), as compared to baseline conditions.

Participants reported both greater satisfaction and higher levels of comfort with each intervention light condition relative to baseline (*p* < 0.001 for SW+, *p* < 0.01 for SW-; see Figure 7, A–B). There was also a main effect for perceived brightness across conditions (*p* < 0.001), with individuals reporting increased brightness with baseline as compared to both intervention workplace lighting conditions (*p* < 0.01 for both SW+ and SW-; Figure 7C). The two interventions were not statistically different from one another on any of these three measures (all *p* ≥ 0.28).

**Figure 8.**
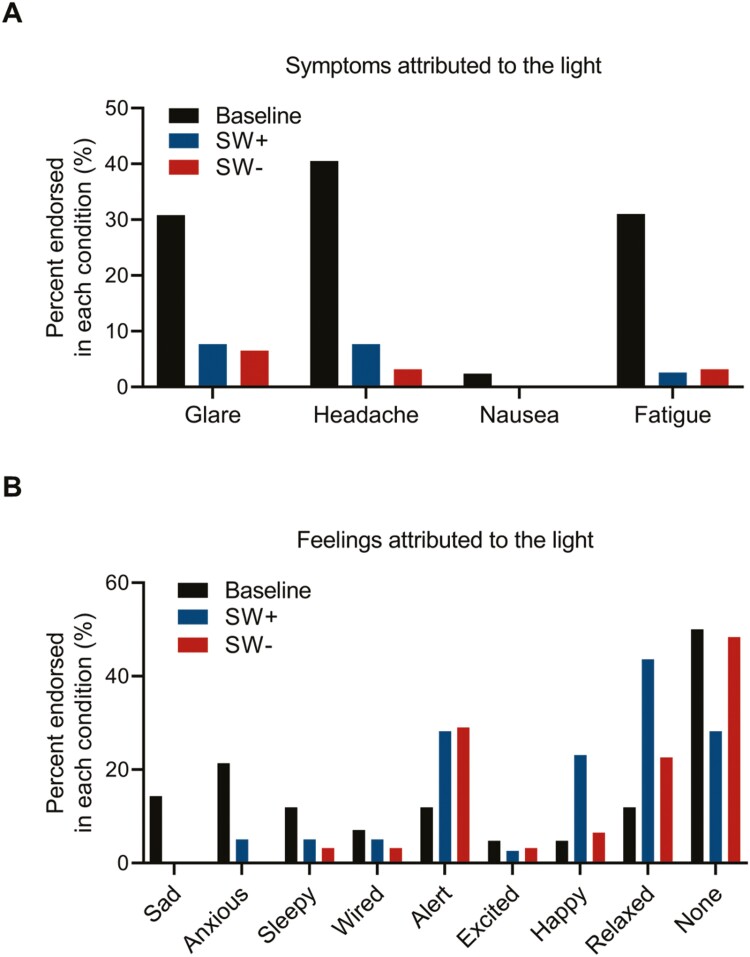
Mood, feelings, and symptoms attributed to the light boxes by condition. Participants endorsed feelings they attributed to the light boxes (sad, anxious, sleepy, wired, alert, excited, happy, relaxed, and none) and any symptoms attributed to the light boxes (glare, headache, nausea, and fatigue), after each condition in the ~weekly (every 8 days) questionnaire for baseline (black), SW+ (blue), and SW- (red). (A) There were more headaches, glare, and fatigue in baseline conditions than in both intervention conditions (both *p* < 0.01, *p* < 0.001, and *p* < 0.001 for headaches, glare, and fatigue, respectively), and (B) fewer people endorsed feeling sad and anxious in both intervention conditions as compared to baseline (all *p* < 0.05). Additionally, more people endorsed a positive mood (happy [*p* < 0.05], relaxed [*p* < 0.01]) in SW+ as compared to baseline.

Fewer overall symptoms were attributed to the lights in the intervention conditions as compared with baseline (X^2^_(2)_ = 22.51, *p* < 0.001). Specifically, the number of individuals reporting glare, headaches, and fatigue was significantly lower in both intervention conditions versus baseline (all *p* ≤ 0.01, see Figure 8A). When asked about mood and feelings attributed to the lights, participants also reported more positive mood in both intervention conditions as compared to baseline (X^2^_(16)_ = 43.65, *p* < 0.001). In particular, fewer people endorsed feeling sad and anxious in the two intervention conditions as compared to baseline (both *p* < 0.01, Figure 8B). Additionally, more people reported feeling happy and relaxed in the SW+ condition relative to baseline (*p* ≤ 0.05), with no differences between SW- and baseline for those two feelings (both *p* > 0.13).

When asked about perceptions of efficacy of the light boxes (final weekly questionnaire, *n* = 29), the majority (55.2%) of participants reported that on-shift alertness was overall better with one or both of the light boxes; most of the rest thought it remained the same (41.4%). Two individuals felt that one of the light boxes impaired alertness (one for SW+ and one for SW-), but in both cases, felt alertness was improved in the opposite lighting condition. The majority of participants endorsed wanting to keep one or more of the light boxes (75.9%), with no clear majority preferring one over the other. A handful of participants reported not knowing if they wanted to keep intervention lights (10.3%), and 13.8% of participants did not want to keep either. Multiple choice options were selected for why individuals did not want to keep the light boxes and included: “I don’t think they worked” (*n* = 5), “I don’t need help with my sleep” (*n* = 1), “I didn’t like them” (*n* = 1), and “Other: I felt more alert during the times they were off and slept better” (*n* = 1). Some examples of positive open-ended comments regarding the light boxes included: “It felt like they really worked. Normally I would be fighting sleep but I was alert the whole time” and “I just felt more alert and focused with those ones.” Some neutral comments included: “They haven’t made any particular difference for me” and “Im (sic) fine with or without them.”

#### Blue-blocking glasses.

In the intervention conditions, participants reported wearing blue-blocking glasses on the majority of work days (83.0% and 79.8% for SW+ and SW-, respectively) and days off (64.9% and 64.4% for SW+ and SW-, respectively). They were primarily worn inside at home before bed (94.6%), for approximately half an hour (mean ± SD = 27.40 ± 19.40 and 29.32 ± 26.48 minutes on work and off days, respectively). The majority (75.9%) reported they were somewhat, fairly, or very easy to use, while 17.2% found them somewhat or fairly difficult, and 6.9% had no opinion (Supplementary Figure S2). Some open-ended comments on ease of use included that the blue-blocking glasses were easy to wear over glasses and “not at all hard to use,” while other comments indicated some discomfort or difficulty in remembering to put them on.

Participants reported that their sleep was the same (69.0%) or better (slightly or much, 31.0%) with the blue-blocking glasses as compared to without. Approximately half (48.3%) of the participants indicated they would be likely (slightly-extremely) to use the blue-blocking glasses in the future, while slightly less than half (44.8%) indicated they would be unlikely to do so, and 6.9% had no opinion. Reasons for discontinuing use included thinking that: it would be difficult to incorporate into schedules (*n* = 5), the blue-blocking glasses do not work (*n* = 5), help with sleep is not needed (*n* = 3), and two open-ended comments, one indicating the yellow tint was problematic for vision, and one indicating the blue-blocking glasses do not work well with a gaming headset. Positive open-ended comments regarding future use included, “They help me sleep,” and “I think they are useful as a natural way for sleeping if one has trouble.”

#### Sleep masks.

In the intervention conditions, participants reported wearing sleep masks on the majority of work days (81.1% and 69.8% for SW+ and SW-, respectively) and days off (79.0% and 67.7% for SW+ and SW-, respectively). More than half of the participants (75.8%) reported they were very, somewhat, or fairly easy to use, while 24.1% found them somewhat, fairly, or very difficult to use (Supplementary Figure S3). Positive open-ended comments on ease of use included, “Easy to put on” and “I kept it by my bed so it made it easy to put on before bed and it’s actually comfortable.” Negative ease of use comments included mentions of the mask not working with a CPAP machine and the mask moving or falling off during sleep.

All participants reported that their sleep was better (slightly or much better, 62.1%) or the same (37.9%) with sleep masks as compared to without. Additionally, the majority of participants (72.4%) indicated they would be likely (slightly-extremely) to use the sleep masks in the future, 6.9% had no opinion, and 20.7% indicated they would be unlikely to use them. Reasons for discontinuing use included discomfort with having something on one’s face while sleeping. Positive open-ended comments regarding future use included, “They help me stay asleep with the sun coming up” and “In my opinion a must for sleeping during the fay (sic).”

## Discussion

Two multi-component lighting interventions that each contained a distinct, spectrally engineered light source were developed, implemented, and studied in night shiftworkers in a high-security watchfloor environment. Both interventions yielded positive benefits; however, the pattern of results was not identical under the two conditions, nor was it always consistent between objective and subjective measures. The primary strength of this study is the intervention design, developed to manipulate the day–night contrast in melanopic EDI without significant alterations in visual stimulation. To that end, the two interventions included white-appearing, near-metameric light boxes of similar photopic intensities yet distinct biological potencies. Many prior lighting countermeasures for shiftworkers have employed high-intensity light (≥1000 lux), which can be hard to tolerate [4, 6, 22, 24–26]. In addition, lighting has often been in the form of off-the-shelf light boxes and/or retrofitted ceiling fixtures that can be challenging to implement in work settings, and can differ considerably in terms of not only spectral quality but also color temperature, intensity, spatial configuration, and other characteristics that may further influence physiological responses [4, 8, 54, 77–81]. Thus, the use of lights that vary across multiple dimensions may conflate findings and lead to differential impacts that are independent of spectral quality per se, and may impact usability as well.

This underscores the importance of a second strength of this study, the sizable implementation component. Measures that assess potential barriers to uptake, as well as other factors related to user experience, offer critical insight into why individuals may or may not choose to utilize these intervention tools in the future [60]. An intervention that is effective but is cumbersome, burdensome, or uncomfortable will not be used, and therefore its efficacy under experimental conditions (wherein individuals are often directly compensated for participation and/or under social pressure to comply) may be of more limited value in practice. Despite the ultimate aim of translation to application, implementation is not commonly assessed in shiftwork studies [58]. Many of the implementation measures used here were adapted from recent work [54], and our selection of intervention tools for this study (e.g. sleep mask style) was informed by those data. Importantly, all components of the intervention here were perceived as easy to use and well-received by participants, even in this population and workplace environment that posed unique challenges.

In contrast to the bright, light-based field studies described above, the photopic lux levels of our light boxes are more similar to the control comparison of many prior reports [6, 24–26, 79, 80, 82, 83]. This is likely at least partly responsible for the relatively lower levels of symptoms (e.g. glare, headache) reported here under intervention conditions. Interestingly, when participants were asked to rate perceived brightness of each condition, they reported the standard, baseline lighting condition as significantly brighter than both interventions, despite being exposed to less light at baseline. Lighting in the baseline condition was actually quite stark, with dark surfaces, fluorescent tube bulbs, and numerous spots of light from all the monitors housed in this relatively small space. The perception of reduced glare, as reported by participants, is likely attributable to the more uniform illumination provided by the addition of intervention lights at the front of the space. Participants also reported desire to continue use, as well as significantly greater levels of comfort and satisfaction, with both lighting interventions, which is interesting given their distinct spectral qualities and effects.

Differential effects were expected given that the two interventions had different biological potencies. We hypothesized that SW+ would elicit broad physiological effects whereas SW- would enhance subjective alertness and PVT performance but minimize sustained alterations in circadian rhythms (e.g. sleep). While short-wavelength light has been shown to be the most potent for stimulating a variety of physiological responses to light [10], limited prior studies have reported alerting effects of short-wavelength-attenuated and longer wavelength light [50, 84]. In this study, sleep, mood, lighting satisfaction, comfort, and symptoms all improved under SW+. Furthermore, improvements in sleep quality extended to days off in this condition, consistent with expected sustained responses to SW+ light. In contrast, alertness, QOL, and both PVT and perceived performance were largely unaffected in this condition.

While perhaps some of the SW+ results are theoretically surprising, previous studies of lighting interventions for shiftworkers have often also yielded inconsistent results with short-wavelength-enriched light [54, 79, 80], whereas studies in the lab tend to be more consistent [85–87]. Ceiling effects for these particular responses may explain the absence of effects in some cases; however, this is unlikely here, as baseline lighting conditions in the current study were not at saturating levels on the dose–response curve for physiological effects that have been characterized [36, 38]. In addition, given many of these outcomes improved from baseline in SW-, there was indeed room for improvement. Despite the hypervigilance required in this operational context, at baseline, participants had decrements in alertness and sleep patterns that are typical of individuals working nonstandard schedules, feeling least alert at the end of the shift, and sleeping less on work days than on days off (Figure 1, Tables 1 and 2) [6, 54, 88, 89]. Some measures that failed to improve, such as performance on the PVT, may be a consequence of insufficient power and/or necessary deviations from our preferred methods of assessment. In the future, other assessments of attention and/or higher-level cognitive function could prove more useful or have greater ecological validity.

Our SW+ condition, but not SW- (or baseline), meets the daytime light exposure levels recommended for optimal health (melanopic EDI > 250 lux) [48]. Yet, in terms of SW-, we found increases in on-shift alertness, coupled with improvements in mood, QOL, perceived performance, lighting satisfaction, comfort, and symptoms, as well as reduced sleep onset latency upon returning home. The decreased sleep onset latency may be due to relatively less impact on the circadian system or increases in homeostatic pressure, as sleep quality was significantly lower in SW- relative to SW+ but with no differences in sleep duration. These findings are generally consistent with limited prior work demonstrating select improvements in many of these outcomes with short-wavelength-attenuated or long-wavelength light [50, 84, 85, 90].

Perhaps the more interesting comparison is to a study that reported similar results using polychromatic “blue-*enriched*” light but with a comparable melanopic content to our “short-wavelength-*attenuate*d” condition (132–150 lux) [49]. Though that was a highly controlled laboratory study employing different methods, it does illustrate the fact that descriptors of spectral quality alone are not sufficient, and highlights the importance of this new metric for comparisons across studies [16, 47]. Unfortunately, many publications to date have not included details about the lighting that are necessary to calculate melanopic EDI, but there has been a recent push in the field to report spectral power distributions and alpha-opic illuminances, and our findings underscore the value of this movement [10, 47, 91].

The increased alertness in the SW- condition is all the more remarkable given the relatively poorer quality sleep and the lower amount of caffeine consumed. While light and caffeine can have potent synergistic benefits [92], caffeine has a long half-life, whereas the alerting effects of light are expected to be relatively acute [32]. Sustainment of the alerting response to light has yet to be fully characterized; however, improvements in alertness reported here did not extend beyond the time on-shift and thus, effects were acute and likely attributable to the SW- light boxes in the work space. Based on these findings and those of others [50], melanopic EDI may not necessarily best reflect the biological potency of light for increasing alertness and/or required values may vary depending on the desired physiological effect. As research advances with this new metric, multiple physiological effects of light should be studied in the same individuals to better understand how potency may vary with light of different spectral qualities across responses.

When considering a night shiftworker’s “day” and “night,” it is both the contrast in light levels, as well as absolute values, which are critical. Many shiftwork field studies manipulate light during only a portion of the shift and/or do not include additional tools for modulating light during off-shift hours. Both the SW+ and SW- interventions likely meet the newly established evening and nighttime recommendations (melanopic EDI < 10 and < 1 lux, respectively) with the added use of blue-blocking glasses and sleep masks [48]. While it is not possible to tease apart the potential contribution of the blue-blocking glasses and sleep masks, they were used in conjunction with both lighting interventions and therefore, the differences in results between the two intervention conditions are likely at least partly attributable to differential effects of the spectral quality of light in the workplace. In addition, the improved sleep quality reported on days off in the SW+ condition may be due to the usage of blue-blocking glasses, use of sleep masks, and/or aftereffects of SW+ on preceding work nights. Prior work has demonstrated efficacy of blue-blocking glasses and sleep masks alone [55, 93–97], so it is reasonable to assume that each piece contributed to the findings observed here. Interestingly, more individuals thought they might use the blue-blocking glasses in the future (48.3%) than thought they helped improve sleep (31.0%), which could be due to additional benefits to using the blue-blocking glasses (reduced glare or stress), or perhaps their high perceived ease of use (75.9%) made individuals more likely to continue to try them though they may not have felt immediately helpful (Supplementary Figure S2). Reasons for not using both tools included the belief that they do not work, or help with sleep is not needed, suggesting additional feedback or pre-study education to explain the reason for usage might increase adherence and/or future uptake. While our choice of product was informed by prior implementation data [54], there is also potential for modifications for those who must wear other face gear (e.g. eye glasses or CPAP), and for options that maximize investment, comfort, and style on an individual basis.

In contrast to blue-blocking glasses and sleep masks, light box exposure did not hinge on participant adherence, though the intensity of the light varied between participants, depending on where in the space-time was spent. This did not likely vary much within individuals across intervention conditions, as participants generally spent the majority of their time on the watchfloor at the same workstation area. Unfortunately, we were unable to reliably assess individual photic exposure patterns despite the use of actigraph watches with built-in photosensors. While the data collection took place within the summer months, all service members were necessarily in uniform during work hours, and the watches were intermittently covered by sleeves. We intended to include photosensors near the head in the direction of gaze, as well as continuous measurement of the environmental light; however, the equipment was not permitted on the watchfloor. Future studies should employ light sensors on a lanyard or another location on the body that is closer to the eye, that remains unobstructed by uniforms or sleeves, and that are positioned in the direction of gaze.

As described in the methods, the high-security nature of the watchfloor posed unique additional challenges, primarily that it precluded use of many of our typical methods of data collection (e.g. unrestricted text message reminders, convenient and accessible tablets, photosensors near the eye and in the direction of gaze, continuous light measurement of this dynamic lighting environment). Additionally, as with all applied work, data collection by participants was secondary to work tasks, therefore there are differences in dataset composition and size between outcomes, which could account for the lack of correspondence between objective and subjective outcomes generally. Yet another contribution to missing data and experimental challenges included the timing of the study relative to the COVID-19 pandemic. Furthermore, we cannot rule out the influence of possible light history effects on our findings. Participants were on staggered rotating schedules, and testing two different lighting conditions within the same individuals necessitated a protocol that spanned a period of ~48 days per individual, and 10 weeks for the whole study (though it was constrained to summer months). We chose to study the SW+ intervention first, as the SW- condition was more exploratory in nature (i.e. potentially alerting with fewer sustained effects). Beginning with the more biologically potent stimulus increased the potential for effects of light history; however, the 16–32 day interval between intervention conditions was likely a sufficient washout period (Figure 4). We further minimized history effects by comparing the results of both interventions to the first baseline whenever possible. Additionally, any light history effects are likely not pronounced, as there was only one finding in either condition on days off. Placebo effects are also possible but do not likely fully account for the findings due to the fact that there were selective improvements in one intervention for some outcomes, and different improvements for the other. Finally, the potential influence of placebo effects was reduced by designing lights that were white-appearing, not significantly brighter than typical indoor lighting, and near-cone metamers.

In summary, this multi-component lighting intervention led to improvements in sleep, alertness, mood, and QOL for night shiftworkers despite the limitations and challenges presented by the high-security setting. By engineering two types of spectrally distinct yet visually similar light boxes, we were able to augment the lighting in a unique work environment to improve sleep, alertness, and mood without compromising visual comfort and satisfaction. While both lighting interventions were well-received and led to improvements, more work is needed to tease apart the various factors contributing to the differences between results. We would have expected that short-wavelength-enriched light would demonstrate more sustained physiological effects compared to short-wavelength-attenuated light, but we did not find that to be true across all measures. Future studies should try to include assessments in less restrictive work environments and different shiftworker populations, as well as incorporate enhanced measures of individual photic exposure patterns (i.e. photosensors near the eye and in the direction of gaze) and circadian phase (e.g. melatonin and/or body temperature profiles). While there is more work to be done in developing light-based applications for shiftworkers, this study demonstrates how spectrally distinct lights hold promise for improving alertness, sleep, and health in more targeted ways.

## Supplementary Material

zpad051_suppl_Supplementary_Tables_S1-S2_Figures_S1-S3

## Data Availability

The data underlying this article will be shared upon reasonable request to the corresponding author, with permission of Uniformed Services University and the Department of Defense.

## DoD Disclaimers

HJF Disclaimer: The contents of this publication are the sole responsibility of the author(s) and do not necessarily reflect the views, opinions, or policies of The Henry M. Jackson Foundation for the Advancement of Military Medicine, Inc. Mention of trade names, commercial products, or organizations does not imply endorsement by the U.S. Government. The authors report no conflicts of interest in this work. USU disclaimer: The opinions and assertions expressed herein are those of the author(s) and do not necessarily reflect the official policy or position of the Uniformed Services University of the Health Sciences (USU) or the Department of Defense (DoD). References to nonfederal entities or products do not constitute or imply a DoD or USU endorsement. This research protocol was reviewed and approved by the USU institutional review board in accordance with all applicable Federal regulations governing the protection of humans in research (protocol# USUHS.2019-016). This work was prepared by a military or civilian employee (GG) of the U.S. Government as part of the individual’s official duties and therefore is in the public domain and does not possess copyright protection (public domain information may be freely distributed and copied; however, as a courtesy it is requested that USU and the author be given an appropriate acknowledgment).
