# Supplementary material for: Hybrid effectiveness-implementation study of two novel spectrally engineered lighting interventions for shiftworkers on a high-security watchfloor
Source: Sleep Adv. 2023 Nov 21;4(1):zpad051. doi: 10.1093/sleepadvances/zpad051 (PMC10710545; doi:10.1093/sleepadvances/zpad051)
Supplement: zpad051_suppl_Supplementary_Tables_S1-S2_Figures_S1-S3 [file zpad051_suppl_supplementary_tables_s1-s2_figures_s1-s3.pdf]

Title

Hybrid effectiveness-implementation study of two novel spectrally-engineered lighting interventions for shiftworkers on a high-security watchfloor

Authors and Affiliations

Sara C. Bessman[1,2], Elizabeth M. Harrison[1,2], Alexandra P. Easterling[1,2], Michelle N. Snider[1,2] Sebastian M.M. Preilipper[1,2], Gena L. Glickman[2]

[1]Henry M. Jackson Foundation for the Advancement of Military Medicine, Inc. (HJF)

[2]Department of Psychiatry, Uniformed Services University of the Health Sciences

Corresponding Authors

Sara C. Bessman

4301 Jones Bridge Road

Bethesda, MD 20814

sara.bessman.ctr@usuhs.edu

Gena L. Glickman

4301 Jones Bridge Road

Bethesda, MD 20814

gena.glickman@usuhs.edu

Supplemental Material

Hybrid effectiveness-implementation study of two novel spectrally-engineered lighting interventions for shiftworkers on a high-security watchfloor. Bessman et al. *Sleep Advances.* 2023.

Table S1. Participant characteristics

	n (%)
Sex	
Male	39 (83.0)
Female	8 (17.0)
Age	
17-24	12 (25.5)
25-29	18 (38.3)
30-39	17 (36.2)
Race/Ethnicity	
Non-Hispanic White	21 (44.7)
Black	6 (12.8)
Hispanic	5 (10.6)
Asian/Pacific Islander	3 (6.4)
Multiracial	12 (25.5)
Marital Status	
Married/Cohabitating	25 (53.2)
Separated/Divorced/Widowed	5 (10.6)
Never Married	17 (36.2)
Paygrade/Rank	
E1-E3	1 (2.2)
E4-E6	40 (88.9)
E7-E9	3 (6.7)
O1-O3	1 (2.2)
Chronotype	
Definitely Morning Type	3 (7.9)
Moderately Morning Type	10 (26.3)
Neither Type	17 (44.7)
Moderately Evening Type	7 (18.4)
Definitely Evening Type	1 (2.6)

E=Enlisted, O=Officer; n=47 for all variables except Rank (n=45) and Chronotype (n=38)

Figure S1

Figure S1. Vector plots of mid-sleep times across conditions for both diary and actigraphy on work days and days off. Each dot represents a single individual's average mid-sleep. Vector line (arrow) points to the mean mid-sleep time for that condition, and line length indicates the degree of clustering of the data points within each condition (baseline [black], SW+ [blue], and SW- [red]). Mid-sleep times were more visually clustered for work days than days off, and centered around ~11:00 on work days and ~6:00 on days off for all conditions and both measures. Watson-Williams tests were performed on each dataset to examine differences by condition; none were statistically significant (all p>0.39; see table S3).

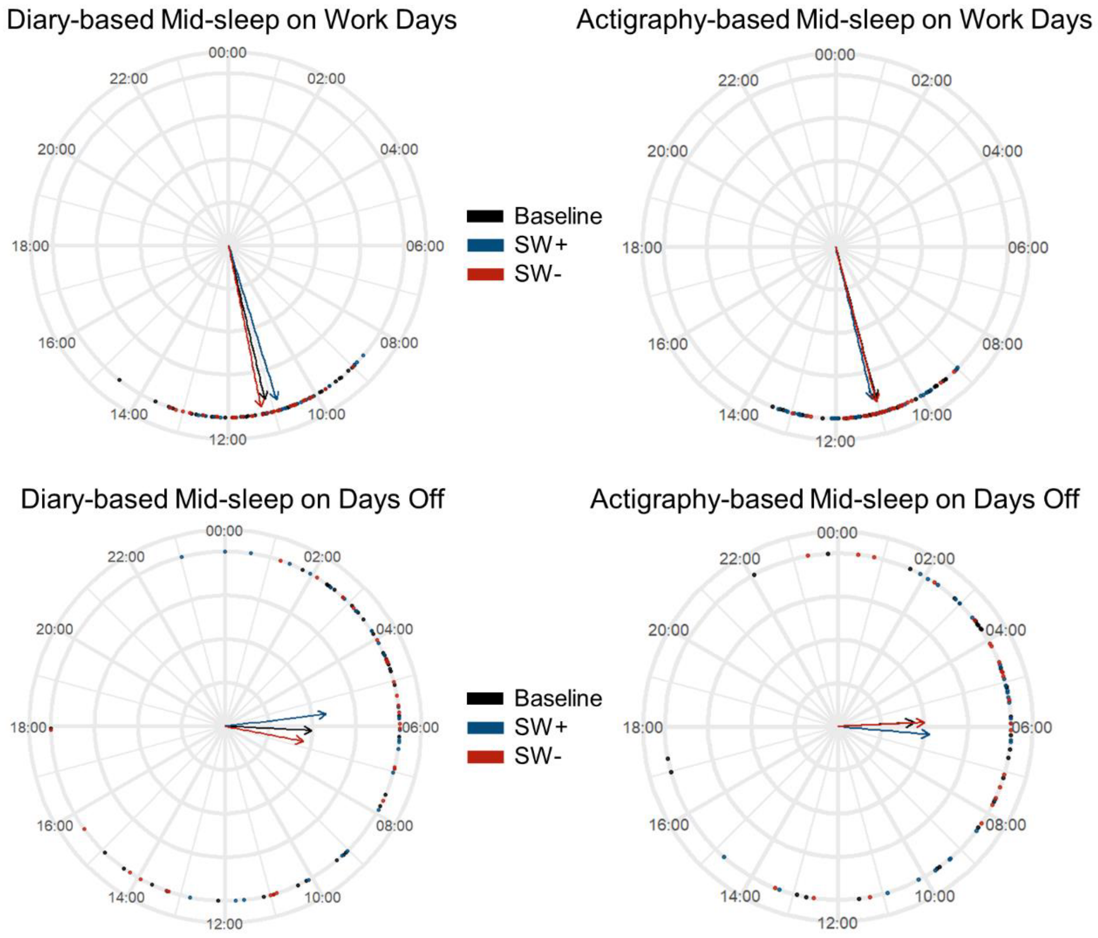

Table S2. Mean clock times and standard deviations for sleep, mid-sleep, and wake times by condition for diary and actigraphy on work days and days off

			Baseline	SW+	SW-	F (df)	p
Work days	Diary	Sleep	7:53 (1:42)	7:36 (1:15)	7:45 (1:27)	0.46 (2,108)	0.63
		Mid	11:10 (1:34)	10:54 (1:21)	11:22 (1:37)	0.96 (2,108)	0.39
		Wake	14:27 (1:39)	14:13 (1:43)	14:46 (1:48)	1.03 (2,108)	0.36
	Actigraphy	Sleep	7:44 (1:51)	7:47 (1:47)	7:35 (1:33)	0.21 (2,100)	0.81
		Mid	11:00 (1:38)	11:06 (1:37)	11:01 (1:25)	0.13 (2,100)	0.88
		Wake	14:15 (1:53)	14:26 (1:56)	14:27 (1:41)	0.08 (2,100)	0.93
Days off	Diary	Sleep	2:15 (4:22)	1:35 (4:02)	2:36 (5:11)	0.13 (2,91)	0.88
		Mid	6:12 (4:25)	5:26 (3:55)	6:36 (5:07)	0.32 (2,91)	0.73
		Wake	10:05 (4:28)	9:23 (3:56)	10:35 (5:01)	0.32 (2,91)	0.73
	Actigraphy	Sleep	1:22 (5:34)	1:57 (4:28)	1:39 (4:31)	0.46 (2,75)	0.64
		Mid	5:49 (4:53)	6:20 (4:17)	5:49 (4:29)	0.24 (2,75)	0.79
		Wake	10:02 (4:28)	10:35 (4:11)	9:54 (4:43)	0.40 (2,75)	0.67

SW+ = short wavelength-enriched intervention. SW- = short wavelength-attenuated intervention. Mid= Mid-sleep Time. N's differ for diary and actigraphy, as well as for work days vs. days off.

Figure S2

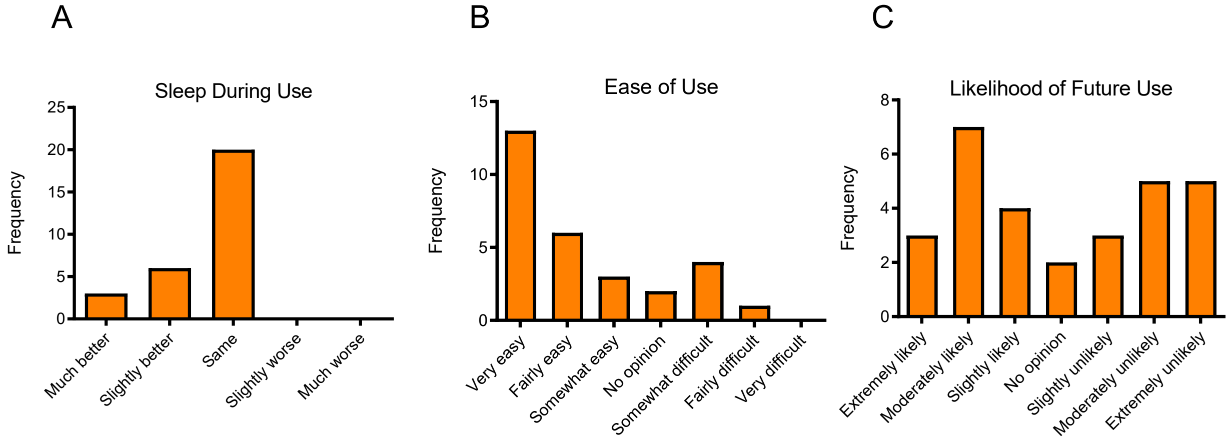

Figure S2. Self-reported sleep and implementation data for the blue-blocking glasses worn during intervention conditions. Participants reported (A) whether their sleep was better, worse, or the same on days they used the blue-blocking glasses, compared to their usual sleep; (B) how easy or difficult it was for them to use the blue-blocking glasses (very easy-very difficult); and (C) how likely they were to continue using the blue-blocking glasses on their own after the intervention ended (extremely likely-extremely unlikely).

Figure S3

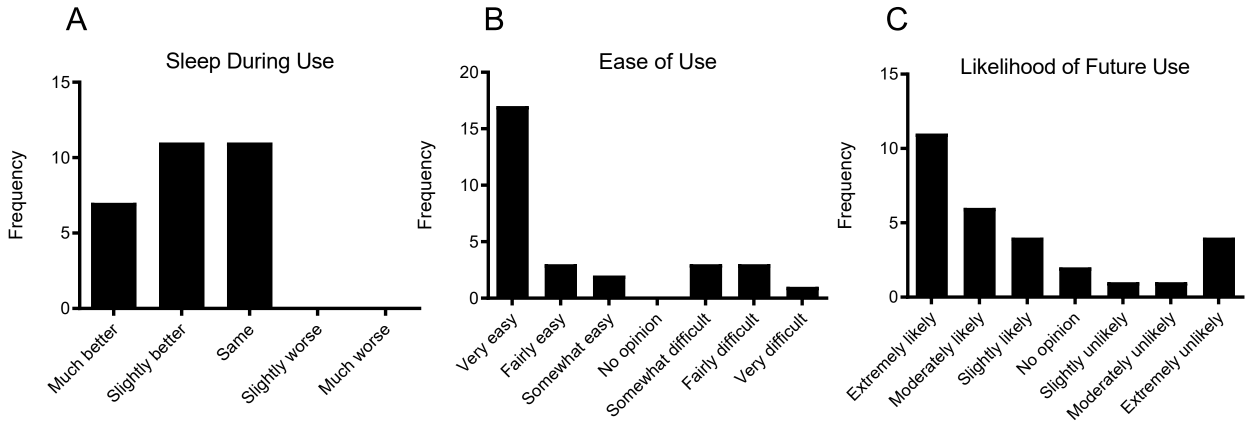

Figure S3. Self-reported sleep and implementation data for the sleep masks worn during intervention conditions. Participants reported (A) whether their sleep was better, worse, or the same on days they used the sleep mask, compared to their usual sleep; (B) how easy or difficult it was for them to use the sleep masks (very easy-very difficult); and (C) how likely they were to continue using the sleep masks glasses on their own after the intervention ended (extremely likely-extremely unlikely).
